# Genital hiatus area and pelvic floor dysfunction symptoms in transgender women after gender-affirming surgery

**DOI:** 10.61622/rbgo/2026rbgo21

**Published:** 2026-03-20

**Authors:** Marina Hazin, Andrea Lemos, Rogerson Andrade, Letícia Gantzel, Leila Barbosa, Caroline Wanderley Souto Ferreira

**Affiliations:** 1 Universidade Federal de Pernambuco Laboratory of Women's Health and Pelvic Floor Department of Physiotherapy Recife PE Brazil Laboratory of Women's Health and Pelvic Floor, Department of Physiotherapy, Universidade Federal de Pernambuco, Recife, PE, Brazil.; 2 Universidade Federal de Pernambuco Hospital de Câncer de Pernambuco Recife PE Brazil Hospital de Câncer de Pernambuco, Universidade Federal de Pernambuco, Recife, PE, Brazil.

**Keywords:** Gender dysphoria, Pelvic floor, Pelvic floor disorders, Gender-affirming surgery, Transgender persons

## Abstract

**Objective:**

To describe genital hiatus area, pelvic floor muscle (PFM) function, pelvic floor dysfunction symptoms and sexual function characteristics of transgender women submitted to gender-affirming surgery.

**Methods:**

Six participants were included in this case series conducted in a referral hospital which treats transgender individuals in Brazil. An individual assessment was performed with sociodemographic variables, clinical symptoms, PFM function, neovaginal depth and genital distances. Two and three-dimensional translabial ultrasound were used to measure hiatal dimensions. The results were shown with absolute and relative frequencies, mean and standard deviation.

**Results:**

The genital hiatus area had an average of 11.09 (SD 3.49) cm² at rest and 14.36 (SD 3.00) cm² during the Valsalva maneuver. The mean distances of clitoris-vagina, genital hiatus and perineal body were 9.30 (SD 1.57) cm, 6.60 (SD 1.43) cm and 4.00 (SD 1.90) cm, respectively. The mean vaginal depth was 9.50 (SD 3.51) cm. Most volunteers had satisfactory PFM strength and tone. Transgender women reported an increase in daytime urinary frequency (100%), nocturia (83.3%), sensation of incomplete evacuation (66.7%), straining during defecation (50%), flatal incontinence (16.6%), dyspareunia (50%) and anodyspareunia (16.6%). There were no reports of urinary or fecal incontinence, vaginal bulging, perineal pain or dysuria.

**Conclusion:**

This is the first study reporting genital hiatus area by translabial ultrasound in transgender women. Adequate levator hiatal area and neovaginal depth were found, preserved PFM tone and strength, preserved urinary and fecal continence, increased daytime urinary frequency, nocturia, sensation of incomplete evacuation, dyspareunia during vaginal sex and moderate vaginal sexual satisfaction.

## Introduction

A transgender person is someone whose gender identity is incongruence with birth assigned sex. The incongruence between the biological genital morphology at birth and the sociocultural attributions of transgender individuals, combined with societal pressures and the pursuit of personal identity, bodily autonomy, and psychological well-being, may lead to the desire for gender transition.^(1)^

This process can occur through different pathways, including social transition, puberty suppression, hormone therapy, and gender-affirming surgeries, depending on each individual's needs, goals, and circumstances. Among the surgical methods, the penoscrotal inversion or penile inversion is the most common gender-affirming surgery for vaginoplasty,^(2,3)^ since it poses low risk of complications and almost no visible scarring. Patients are generally satisfied with neovagina functionality and esthetic appearance following surgery.^(4)^

Despite the benefits, this surgery may have adverse effects, including tightening of the introitus and neovaginal canal.^(5)^ The procedures to form neovaginal cavity can modify the genital hiatus area. The 3D ultrasound evaluation in cisgender women shows that those with larger area of genital hiatus in Valsalva maneuver may report symptoms of pelvic organ prolapse, while those with smaller area may report stress UI.^(6)^ The musculotendinous structures involved in this surgical procedure may affect pelvic floor muscles (PFM) function and favor the appearance of pelvic floor dysfunction symptoms, such as urinary incontinence (UI), dyspareunia and voiding dysfunction.^(5,7)^

Some studies describe postoperative pelvic floor dysfunction symptoms in transgender women undergoing gender-affirming surgery but studies on the implications of surgery on PFM function are scarce.^(7-10)^ Moreover, no studies evaluating genital hiatus area in this population were identified. Finally, the aim of this study was to describe genital hiatus area through translabial ultrasound, PFM function, pelvic floor dysfunction symptoms and sexual function characteristics of transgender women submitted to gender-affirming surgery.

## Methods

This case series study was conducted in the Laboratory of Women's Health and Pelvic Floor, Department of Physiotherapy, *Universidade Federal de Pernambuco*, (UFPE) Brazil.

The volunteers were followed in a referral hospital and participated in a program governed by the laws of the Unified Health System (*Sistema Unico de Saúde* - SUS)/Brazil, which provides care for transgender and gender-diverse individuals. In this program, pre and post-surgery follow-up is conducted by a multidisciplinary team composed of doctors (urologist, gynecologist, endocrinologist and psychiatrist), nurses, psychologists, physiotherapist and social workers.

The inclusion criteria were transgender women, aged 21 years or older, who had undergone gender-affirming surgery. The exclusion criteria were transgender women with a history of neuromuscular and conjunctive tissue disease. All transgender women (n = 10) who underwent gender-affirming surgery in Hospital das Clínicas of Pernambuco were contacted by telephone. Six participants (age 22 to 40 years) attended the hospital for evaluation, met the eligibility criteria and were included. Among the six participants, participant number three was not sexually active before study. All volunteers gave their written informed consent.

The surgery was performed by a trained surgeon team, using the penoscrotal inversion technique. The steps include (1) scrotal incision; (2) removal of both testicles; (3) perineal body incision to create space for the neovagina after resection of the urogenital diaphragm; (4) circular cut around the skin of the body of penis under the glans and separation of the penile skin from the corpus cavernosum and spongiosum; (5) separation of the urethra (corpus spongiosum) from the corpus cavernosum and resection to reduce its size; (6) preservation of one part of the glans and dissection of the neurovascular bundle of the corpus cavernosum; (7) bilateral dissection of the corpus cavernosum proximal to the pubic symphysis; (8) inversion of penile skin and closing of its distal part with insertion of the skin into the cavity formed; (9) skin incision to allow passage of the clitoris (formed from the glans penis) and the urethra; (10) adjustment of the labia majora, made from the scrotal skin.^(11)^

Initially, the volunteer answered an individual form, consisting of sociodemographic variables, clinical symptoms, sexual activity and sexual satisfaction. Urinary, fecal and prolapse symptoms were defined according to International Urogynecological Association and International Continence Society (IUGA/ICS) guidelines.^(12,13)^

Sexual satisfaction was assessed using the Visual Analog Scale (VAS), whose score varied from 0 to 10, 0 being the absence of satisfaction and 10 maximum satisfaction.^(14)^ Vaginal and anal dyspareunia were also measured by the VAS, on a scale of 0 to 10, 0 being the total absence of pain and 10 the most extreme pain possible.^(15)^ The Female Sexual Function Index (FSFI) was not used, as the instrument has not been validated for the transgender female population.

Two and three-dimensional translabial ultrasound (TLUS) were performed in the supine position, after bladder emptying using a GE Voluson E6 System (GE Medical Systems, Zipf, Austria), with a volumetric transducer Ultrasound (US). The main outcome was hiatal dimension, which was measured in the axial plane at the plane of minimal hiatal dimensions, as previously described. The hiatal area of the levator ani muscle, delimited by the puborectalis muscle, symphysis pubis (SP) and inferior pubic ramus, was assessed both at rest and its excursion during the Valsalva maneuver (three repetitions) to evaluate its variations, avoiding coactivation of the levator ani.^(16)^

Neovaginal depth and the distances between the clitoris and vagina, the urethra and vagina [(genital hiatus (GH)] and the vagina and anus [(perineal body (PB)] were measured through a POP-Q Ruler, numbered from 0 to 150 millimeters (mm), covered by a condom.^(17)^ PFM function was assessed using the PERFECT scheme and power was measured according to modified Oxford scale.^(18)^

The results were shown in figures and tables, with absolute and relative frequencies, mean and standard deviation (SD). Descriptive data analysis was conducted using the Statistical Package for the Social Sciences (SPSS) 21.0 software for Windows.

This study was approved by the UFPE Research Ethics Committee (2838691 – CAAE 57439116.9.0000.5208), it followed the principles of the Declaration of Helsinki and recommendations of the National Health Council (Resolution 466/12) and complied with the guidelines recommended by STROBE.

## Results

**Figure 1:**
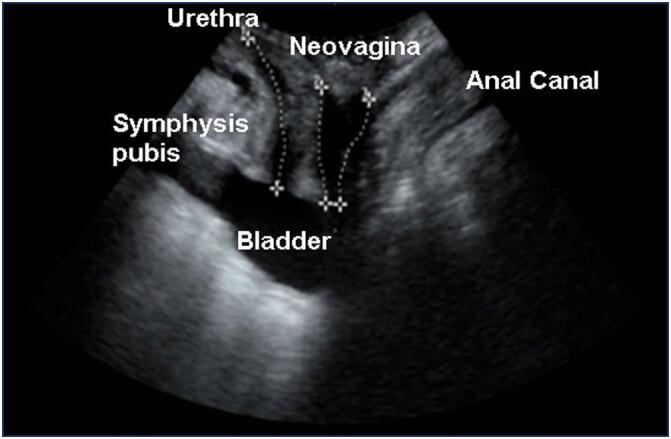
Transperineal ultrasound image in the mid-sagittal plane during Valsalva maneuver in transgender women submitted to gender-affirming surgery

**Figure 2:**
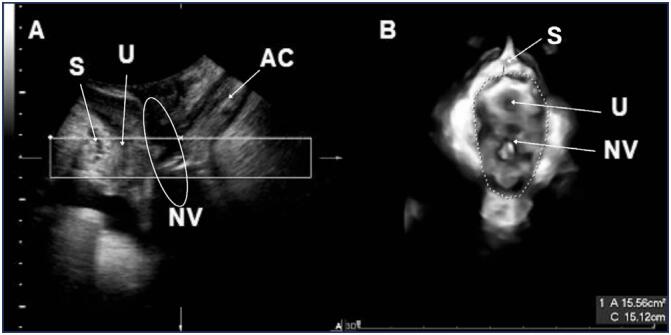
Transperineal ultrasound image to measure the genital hiatus area during the Valsalva maneuver in transgender women submitted to gender-affirming surgery. Pernambuco, Brazil, 2017. A. Image in the mid-sagittal plane to identify pubis symphysis (S), urethra (U), neovagina (NV) and anal canal (AC). B. Axial view of the genital hiatus area during the Valsalva maneuver to identify urethra (U) and neovagina (NV)

Six transgender women, with a mean age of 32.00 (SD 7.07; minimum 22; maximum 40) years, body mass index of 23.63 (SD 3.69; ranging from 17.99 to 29.51) Kg/m^2^, mean time of hormone therapy of 12.5 (SD 8.3; ranging from 9 to 25) years and mean time since surgery of 16.5 (SD 7.18; ranging from 11 to 30) months, took part in this study. The mid-sagittal view of transperineal ultrasonographic imaging exhibits the neovagina (Figure 1) and the axial view shows the genital hiatus area (Figure 2), which indicated an average of 11.09 (SD 3.49) cm² at rest (HAR), 14.36 (SD 3.00) cm² during the Valsalva maneuver (HAV) and 3.27 (SD 2.05) cm² the difference between HAV and HAR (Table 1).

**Table 1:** Measurement of genital hiatus area; clitoris-vagina, urethra-vagina and vagina-anus distances; neovaginal depth and pelvic floor muscle function (according to PERFECT scheme) of transgender women submitted to gender-affirming surgery

Variables		Volunteer					
		1	2	3	4	5	6
HAR (cm²)		12.36	10.18	8.9	9.06	8.41	17.61
HAV (cm²)		15.4	14.08	15.56	10.18	12.1	18.82
Δ = HAV - HAR (cm²)		3.04	3.90	6.66	1.12	3.69	1.21
Clitoris-vagina distance (cm)		8	8.5	11	-	11	8
Urethra-vagina distance (cm)		6.5	5.5	9	-	6.5	5.5
Vagina-anus distance (cm)		7	3	4.5	-	2	3.5
Neovaginal depth (cm)		5	9	8	8	15	12
PFM function							
	Power	5	5	5	4	3	4
	Endurance (s)	8	2	4	3	8	0
	Repetitions	10	0	9	8	8	0
	Fast	10	10	10	10	10	7

Footnotes:

* participant refused to continue the assessment; "-" refers to missing data. cm: centimeter; HAR: hiatus area at rest; HAV: hiatus area during the Valsalva maneuver; PFM: pelvic floor muscles

The mean distances of clitoris-vagina, GH and PB were 9.30 (SD 1.57) cm, 6.60 (SD 1.43) cm and 4.00 (SD 1.90) cm, respectively, and the mean neovaginal depth was 9.50 (SD 3.51) cm (Table 1). All participants were able to voluntarily contract PFM, one had difficulty to relax PFM, one had increased tension on the levator ani and one had difficulty to coordinate PFM contraction with breathing. Most volunteers had satisfactory PFM strength, with a predominance of fast fiber contractions activation (Table 1).

Regarding pelvic floor dysfunction symptoms, all transgender women reported daytime urinary frequency between 8 and 12 times, 83.3% referred nocturia, 66.7% had sensation of incomplete evacuation, 50% had straining during defecation and 16.6% complained of flatal incontinence. There were no reports of urinary or fecal incontinence. The volunteers of present study reported no vaginal bulging, perineal pain or dysuria, but three had dyspareunia (mean 2.8; SD 3.3) and one anodyspareunia. The participants who referred pain during sexual intercourse had lower anal (mean 8.3; SD 2.4) and vaginal sexual satisfaction (mean 6.6; SD 4.2). The clinical data on sexual, urinary, fecal and prolapse symptoms are described in table 2.

**Table 2:** Clinical data of sex satisfaction and pelvic floor dysfunction symptoms in transgender women submitted to gender-affirming surgery

Variables	Volunteer					
	1	2	3*	4	5 **	6
Vaginal intercourse satisfaction (VAS *)	0	5	-	8	10	10
Anal intercourse satisfaction (VAS *)	10	5	-	8	-	10
Dyspareunia (VAS **)	8	2	-	4	0	0
Anodyspareunia (VAS **)	0	2	-	0	-	0
Perineal pain	No	No	No	No	No	No
Dysuria	No	No	No	No	No	No
Vaginal bulging	No	No	No	No	No	No
Increased daytime urinary frequency	Yes	Yes	Yes	Yes	Yes	Yes
Nocturia	Yes	Yes	Yes	No	Yes	Yes
Nocturnal enuresis	No	No	No	No	No	No
SUI or UUI	No	No	No	No	No	No
Fecal incontinence	No	No	No	No	No	No
Flatal incontinence	No	No	No	Yes	No	No
Straining to defecate	No	No	Yes	Yes	No	Yes
Sensation of incomplete evacuation	Yes	Yes	Yes	Yes	No	No

Footnotes:

* participant had no vaginal or anal sexual intercourse;

** participant had no anal sexual intercourse; * 0 = very unsatisfied, 10 = very satisfied; ** 0 = no pain, 10 = extremely painful; "-" refers to missing data. s: second; SUI: stress urinary incontinence; UUI: urgency urinary incontinence; VAS: visual analog scale

## Discussion

The findings of the present study show a wide variability in hiatal area of the puborectalis muscle. To our knowledge no studies to assess hiatus area in transgender women undergoing gender-affirming surgery were found. In a study with nulliparous women, the hiatal area of the levator ani at rest and during Valsalva maneuver was 10.62cm² and 11.95cm², respectively.^(19)^ According to literature, a hiatal area greater than 25cm² during the Valsalva maneuver may indicate a laceration or avulsion of the puborectalis muscle.^(20)^ Therefore, transgender women undergoing gender-affirming surgery presented a hiatal area during the Valsalva maneuver within the expected normality for cisgender women, which may suggest that there was no involvement of the puborectalis muscle during the surgical procedure.

In the present study, variability in GH and PB measurements was also observed. A study with cisgender women found that those with pelvic organ prolapse (POP) stage 0 or 1 had a GH distance of 3.59 (SD 1.3) cm and that this distance increases with the severity of the prolapse. Regards to PB measurement, these authors identified an average of 3.34 (SD 0.9) cm for stage 0 or 1 POP cisgender women, with a statistical increase in PB distance only for stage 2 POP, but this increase was not clinically significant (less than 1cm).^(21)^

The variability observed in the hiatal area of the puborectalis muscle, as well as in the measurements of the GH and PB in this study, may be related to preoperative anatomical factors, the manipulation of muscular tissues during surgery, the suturing techniques used, and the postoperative recovery process, including healing and muscle tone.

When compared with data on cisgender women reported in literature, transgender women in the present study exhibited a greater GH measurement and a similar hiatal area during the Valsalva maneuver. The discrepancy in GH area may occur due to the anatomical difference between biological sexes and to the non-standardization of these measures during surgery, since the skin incision to allow passage of the urethra and neovagina is performed according to the specificity of each patient, such as the formation of the neovaginal canal in the retroprostatic space, which possibly places neovaginal introitus at a greater distance in transgender women after surgery.^(22)^

Transgender women in our study exhibited an average neovaginal depth of 9.5cm (range 5 to 15cm). In a study conducted with 100 transgender women submitted to penoscrotal inversion technique, the mean neovaginal depth was 13.8 (SD 1.4) cm right after surgery and 11.4 (SD 2.5) cm 1 year after surgery.^(4)^ Neovaginal depth in this technique depends on the amount of penile skin available.^(3)^ Furthermore, the use of vaginal dilators is important to maintain or increase the size of the canal.^(4)^

With regard to PFM function, all participants were able to voluntarily contract PFM, one had difficult to voluntarily relax PFM due to increased tension, most of them showed satisfactory PFM strength (minimum of 3, according to the Oxford scale) and nearly all had a great number of fast contractions. In a study conducted with 50 participants who underwent gender-affirming vaginoplasty between the years 2016 and 2018, 18 participants (36%) had PFM dysfunction (considered as poorly coordinated contraction–relaxation or muscle weakness). The authors do not describe the method used to assess PFM strength and do not report the assessment of sustained and fast contraction muscle fibers.^(10)^

In penoscrotal inversion surgery the perineum and PFM are dissected to create a functional neovaginal space between the rectum and urethra, prostate and bladder.^(22,23)^ Although this gender-affirming surgery leads to significant changes in pelvic floor morphology, including superficial and deep PFM dissection,^(10,24)^ in this case series, no major impairment of PFM function was observed.

Three volunteers of the present study reported dyspareunia (mean 2.8; SD 3.3) and impairment of vaginal sexual satisfaction (mean 6.6; SD 4.2), although exhibited adequate neovaginal depth. This finding was consistent with a study that evaluated 40 transgender women, 6 months after vaginoplasty, who referred an average pain during masturbation/intercourse of 2.33 (SD 2.89) and a mean satisfaction with sexual intercourse of 6.70 (SD 2.03), both measured by VAS.^(25)^

Pain during neovaginal intercourse may be associated with a lack of natural lubrication and possible fibrosis in the neovaginal canal. Moreover, satisfaction with sexual function and results of surgery may be correlated with neovaginal depth, pain and loss of sensation.^(9)^ Due to the subjective nature of satisfaction and since the transgender population suffers from anxiety and depression,^(26)^ it is important to underscore that not only morphological questions influence the sexual satisfaction of post-gender-affirming surgery women.

Although transgender women in the present study reported an increase in daytime and nighttime voiding frequency, there was no report of urinary or fecal leakage and POP symptoms. Furthermore, part of the sample reported evacuation symptoms. Evidence shows that pelvic floor dysfunction symptoms are reported by transgender women after vaginoplasty.^(5,7,10)^ A study carried out with 50 post-surgery transgender women identified that 28% reported urinary dysfunction and 22% bowel dysfunction.^(10)^ Among the hypotheses for the appearance of symptoms of pelvic floor dysfunction, one would be that the surgical procedure to create the neovaginal space, by the dissection of the PFM, could change the bladder position, as well as cause nerve damage.^(7)^

In Brazil, there are few hospitals specialized in providing care for transgender individuals, as well as a limited number of scientific publications on the subject. A study conducted with 26 Brazilian transgender women who had undergone gender-affirming surgery showed that those who reported sexual activity in the previous four weeks were sexually functional. Additionally, 15.4% presented with urinary incontinence, which had started prior to the surgery, and overall, the participants reported good quality of life.^(27)^

Although all participants who underwent gender-affirming surgery were contacted by telephone, only six attended for evaluation. This was considered a limitation of the study. Other limitations were that preoperative evaluation was not performed, and a validated questionnaire was not used to assess sexual function. Therefore, the results should be considered exploratory and require validation in larger cohorts. As a strength it could be highlighted that, as far as we know, this is the first study to measure the genital hiatus area and show an image of the neovagina, of post gender-affirming surgery transgender women, by translabial ultrasonography.

Future studies should evaluate pelvic floor dysfunction symptoms before and after surgery, as well as to verify mobility of pelvic organs over the years. Furthermore, to suggest reference parameters for GH, PB and hiatus genital area for this population, as there are differences in external genitalia and in the positioning of pelvic organs between cis and trans women.

Regarding recommendations for clinical practice, the findings of this study suggest that penoscrotal inversion surgery may not substantially affect the levator ani muscle function and that when it does occur, that it can be solved by physiotherapy treatment. Therefore, health professionals should evaluate pelvic floor dysfunctional symptoms, as soon as possible, to monitor and refer for early treatment, if necessary. So, despite being invasive, the surgery seems to be safe and has minimal repercussions that can be dealt with over time in the cases where they occur.

## Conclusion

Transgender women who submitted to gender-affirming surgery exhibited adequate levator hiatal area and neovaginal depth, preserved PFM tone and strength, increased daytime urinary frequency, nocturia, sensation of incomplete evacuation, straining during defecation, preserved urinary and fecal continence, dyspareunia during vaginal sex and moderate vaginal sexual satisfaction.

## Data Availability

The research data are described in the article presented.

## References

[1] Beek TF, Cohen-Kettenis PT, Kreukels BP (2016). Gender incongruence/gender dysphoria and its classification history. Int Rev Psychiatry.

[2] Horbach SE, Bouman MB, Smit JM, Özer M, Buncamper ME, Mullender MG (2015). Outcome of vaginoplasty in male-to-female transgenders: a systematic review of surgical techniques. J Sex Med.

[3] Stowell JT, Grimstad FW, Kirkpatrick DL, Brown ER, Santucci RA, Crane C (2019). Imaging findings in transgender patients after gender-affirming surgery. Radiographics.

[4] Buncamper ME, van der Sluis WB, de Vries M, Witte BI, Bouman MB, Mullender MG (2017). Penile inversion vaginoplasty with or without additional full-thickness skin graft. Plast Reconstr Surg.

[5] Rossi R, Hintz F, Krege S, Rubben H, Vom Dorp F (2012). Gender reassignment surgery - a 13 year review of surgical outcomes. Int Braz J Urol.

[6] Albrich SB, Welker K, Wolpert B, Steetskamp J, Porta S, Hasenburg A (2017). How common is ballooning? Hiatal area on 3D transperineal ultrasound in urogynecological patients and its association with lower urinary tract symptoms. Arch Gynecol Obstet.

[7] Kuhn A, Santi A, Birkhäuser M (2011). Vaginal prolapse, pelvic floor function, and related symptoms 16 years after sex reassignment surgery in transsexuals. Fertil Steril.

[8] Manrique OJ, Adabi K, Huang TC, Jorge-Martinez J, Meihofer LE, Brassard P (2019). Assessment of pelvic floor anatomy for male-to-female vaginoplasty and the role of physical therapy on functional and patient-reported outcomes. Ann Plast Surg.

[9] Massie JP, Morrison SD, Van Maasdam J, Satterwhite T (2018). Predictors of patient satisfaction and postoperative complications in penile inversion vaginoplasty. Plast Reconstr Surg.

[10] Jiang DD, Gallagher S, Burchill L, Berli J, Dugi D (2019). Implementation of a pelvic floor physical therapy program for transgender women undergoing gender-affirming vaginoplasty. Obstet Gynecol.

[11] Krempasky C, Grimstad FW, Harris M, Locks RT (2021). Feminizing gender-affirming surgery. J Gynecol Surg.

[12] Haylen BT, de Ridder D, Freeman RM, Swift SE, Berghmans B, Lee J (2010). An International Urogynecological Association (IUGA)/International Continence Society (ICS) joint report on the terminology for female pelvic floor dysfunction. Int Urogynecol J.

[13] Sultan AH, Monga A, Lee J, Emmanuel A, Norton C, Santoro G (2017). An International Urogynecological Association (IUGA)/International Continence Society (ICS) joint report on the terminology for female anorectal dysfunction. Int Urogynecol J.

[14] Koparal MY, Bulut EC, Çetin S, Onaran M, Şen I (2023). Is a one-question visual analog scale a screening tool that can be used to assess female sexual dysfunction before implementing a female sexual function index?. J Urol Surg.

[15] Hurt K, Zahalka F, Halaska M, Rakovicova I, Rakovic J, Cmelinsky V (2021). Extracorporeal shock wave therapy for treating dyspareunia: a prospective, randomized, double-blind, placebo-controlled study. Ann Phys Rehabil Med.

[16] Dietz HP, Bø K, Berghmans B, Mørkved S, Van Kampen M (2007). Evidence-based physical therapy for the pelvic floor: bridging science and clinical practice.

[17] Bump RC, Mattiasson A, Bø K, Brubaker LP, DeLancey JO, Klarskov P (1996). The standardization of terminology of female pelvic organ prolapse and pelvic floor dysfunction. Am J Obstet Gynecol.

[18] Laycock J, Jerwood D (2001). Pelvic floor muscle assessment: the PERFECT scheme. Physiotherapy.

[19] Nesbitt-Hawes EM, Dietz HP, Abbott JA (2018). Morphometry of the nulliparous pelvic floor. Ultrasound Obstet Gynecol.

[20] Dietz HP, Shek C, De Leon J, Steensma AB (2008). Ballooning of the levator hiatus. Ultrasound Obstet Gynecol.

[21] Dunivan GC, Lyons KE, Jeppson PC, Ninivaggio CS, Komesu YM, Alba FM (2016). Pelvic organ prolapse stage and the relationship to genital hiatus and perineal body measurements. Female Pelvic Med Reconstr Surg.

[22] Chen ML, Reyblat P, Poh MM, Chi AC (2019). Overview of surgical techniques in gender-affirming genital surgery. Transl Androl Urol.

[23] Hassan O, Sun D, Jha P (2021). Imaging in gender affirmation surgery. Curr Urol Rep.

[24] Bryson C, Honig SC (2019). Genitourinary complications of gender-affirming surgery. Curr Urol Rep.

[25] Zavlin D, Schaff J, Lellé JD, Jubbal KT, Herschbach P, Henrich G (2018). Male-to-female sex reassignment surgery using the combined vaginoplasty technique: satisfaction of transgender patients with aesthetic, functional, and sexual outcomes. Aesthetic Plast Surg.

[26] Budge SL, Adelson JL, Howard KA (2013). Anxiety and depression in transgender individuals: the roles of transition status, loss, social support, and coping. J Consult Clin Psychol.

[27] Jardim LM, Cerentini TM, Lobato MIR, Costa AB, Silva DC, Schwarz K (2022). Sexual function and quality of life in Brazilian transgender women following gender-affirming surgery: a cross-sectional study. Int J Environ Res Public Health.

